# Relationship between childhood physical abuse and clinical severity of treatment-resistant depression in a geriatric population

**DOI:** 10.1371/journal.pone.0250148

**Published:** 2021-04-20

**Authors:** Antoine Yrondi, Christophe Arbus, Djamila Bennabi, Thierry D’Amato, Frank Bellivier, Thierry Bougerol, Vincent Camus, Philippe Courtet, Olivier Doumy, Jean-Baptiste Genty, Jérôme Holtzmann, Mathilde Horn, Christophe Lancon, Marion Leboyer, Pierre-Michel Llorca, Julia Maruani, Rémi Moirand, Fanny Molière, Jean Petrucci, Raphaelle Richieri, Ludovic Samalin, Florian Stephan, Guillaume Vaiva, Michel Walter, FondaMental Advanced Centres of Expertise in Resistant Depression (FACE-DR) Collaborators, Emmanuel Haffen, Bruno Aouizerate, Wissam El-Hage

**Affiliations:** 1 Fondation FondaMental, Creteil, France; 2 Service de Psychiatrie et de Psychologie Médicale de l’adulte (Department of Psychiatry and Adult Medical Psychology), Centre Expert Dépression Résistante FondaMental (FondaMental Advanced Centre of Expertise in Resistant Depression, CHU de Toulouse (University Hospital Centre), Hôpital Purpan, ToNIC Toulouse NeuroImaging Centre, Université de Toulouse (Toulouse University), INSERM, UPS, Toulouse, France; 3 Service de Psychiatrie, Centre Expert Dépression Résistante FondaMental, CIC-1431 INSERM, CHU de Besançon, Université de Bourgogne Franche Comté, Besançon, France; 4 INSERM U1028; CNRS UMR5292; University Lyon 1, Villeurbanne, F-69000, France; Lyon Neuroscience Research Centre; Psychiatric Disorders: From Resistance to Response ΨR2 Team; Centre Hospitalier Le Vinatier (Hospital Centre); Bron, France; 5 AP-HP, GH Saint-Louis—Lariboisière—Fernand Widal, Pôle Neurosciences Tête et Cou (Head and Neck Neurosciences Cluster), University Paris Diderot, Paris, France; 6 Université Grenoble Alpes, Inserm, U1216, CHU Grenoble Alpes, Grenoble Institut Neurosciences (Institute of Neurosciences), Grenoble, France; 7 U1253, iBrain, CIC1415, Inserm, CHRU de Tours (Regional University Hospital Centre), Université de Tours, Tours, France; 8 Department of Emergency Psychiatry and Acute Care, CHU Montpellier, INSERM U1061, Montpellier University, Montpellier, France; 9 Pôle de Psychiatrie Générale et Universitaire (General and University Psychiatry Cluster), Centre Expert Dépression Résistante FondaMental, CH Charles Perrens, Bordeaux, Laboratoire Nutrition et Neurobiologie intégrée (Integrated Nutrition and Neurobiology Laboratory) (UMR INRAE 1286), Université de Bordeaux (Bordeaux University), Bordeaux, France; 10 Université Paris-Est, UMR_S955, UPEC, Créteil, France Inserm, U955, Equipe 15 Psychiatrie génétique (Team 15 Genetic Psychiatry), Créteil, France AP-HP, Hôpital H. Mondor-A. Chenevier, Pôle de psychiatrie (Psychiatry Cluster), Créteil, France Fondation FondaMental, Fondation de Cooperation Scientifique (Scientific Cooperation Foundation), Créteil, France; 11 Service de Psychiatrie adulte (Department of Adult Psychiatry), Centre Expert Dépression Résistante FondaMental, CHRU de Lille, Hôpital Fontan 1, Lille, France; 12 Pôle Psychiatrie, Centre Expert Dépression Résistante FondaMental, CHU La Conception, Marseille, France; 13 Department of Psychiatry, CHU Clermont-Ferrand, University of Clermont Auvergne, Clermont-Ferrand, France; 14 Service Hospitalo-Universitaire de Psychiatrie Générale et de Réhabilitation Psycho Sociale 29G01 et 29G02 (University Hospital Department of General Psychiatry and Psychosocial Rehabilitation), Centre Expert Depression Résistante FondaMental, CHRU de Brest, Hôpital de Bohars, Brest, France; 15 Centre National de Ressources et Résilience pour les psychotraumatisme (National Resilience and Resources Centre for Psychological Trauma), Lille, France; Universita degli Studi Europea di Roma, ITALY

## Abstract

**Introduction:**

We assessed the correlation between childhood maltreatment (CM) and severity of depression in an elderly unipolar Treatment-Resistant Depression (TRD) sample.

**Methods:**

Patients were enrolled from a longitudinal cohort (FACE-DR) of the French Network of Expert TRD Centres.

**Results:**

Our sample included 96 patients (33% of the overall cohort) aged 60 years or above, with a mean age of 67.2 (SD = 5.7). The majority of the patients were female (62.5%). The Montgomery and Asberg Depression Rating Scale (MADRS) and Quick Inventory Depression Scale-Self Report (QIDS-SR) mean scores were high, 28.2 (SD = 7.49) [MADRS score range: 0–60; moderate severity≥20, high severity≥35] and 16.5 (SD = 4.94) [IDS-SR score range: 0–27; moderate severity≥11, high severity≥16], respectively. Mean self-esteem scores were 22.47 (SD = 6.26) [range 0–30]. In an age- and sex-adjusted model, we found a positive correlation between childhood trauma (CTQ scores) and depressive symptom severity [MADRS (β = 0.274; p = 0.07) and QIDS-SR (β = 0.302; p = 0.005) scores]. We detected a statistically significant correlation between physical abuse and depressive symptom severity [MADRS (β = 0.304; p = 0.03) and QIDS-SR (β = 0.362; p = 0.005) scores]. We did not observe any significant correlation between other types of trauma and depressive symptom severity. We showed that self-esteem (Rosenberg scale) mediated the effect of physical abuse (PA) on the intensity of depressive symptoms [MADRS: *b* = 0.318, 95% *BCa C*.*I*. [0.07, 0.62]; QIDS-SR: *b* = 0.177, 95% *BCa C*.*I*. [0.04, 0.37]]. Preacher & Kelly’s Kappa Squared values of 19.1% (*k*^*2*^ = 0.191) and 16% (*k*^*2*^ = 0.16), respectively for the two scales, indicate a moderate effect.

**Conclusion:**

To our knowledge, this is the first study conducted in a geriatric TRD population documenting an association between childhood trauma (mainly relating to PA) and the intensity of depressive symptoms.

## Introduction

Depression is a very common disorder in the elderly, with a prevalence of up to 30% [1].

Geriatric depression is currently defined as the occurrence of depressive episodes in the elderly, although the age at onset is critical, with early- (EOD; first episode before the age of 60) or late-onset depression (LOD; first episode after the age of 60). Some differences have been reported between EOD and LOD [2–4]. For instance, personality abnormalities and a family history of psychiatric illness were significantly more common in EOD [3, 4]. However, when the severity, phenomenology, history of previous episodes, and neuropsychological performance were considered, no difference was found between EOD and LOD in elderly people [3, 4]. As shown by van Krugten et al., an older age, a high severity, a poor treatment response and childhood trauma should be sought due to their association with need for highly specialized Major Depressive Disorder (MDD) care [5].

Half of elderly depressed adults report some form of childhood abuse [6]. Childhood mistreatment is associated with the development of geriatric depression, albeit more frequently in EOD [1, 6]. There is also a link between childhood abuse and EOD in older populations [7]. In addition, the relationship between childhood maltreatment and geriatric depression (EOD or LOD) is mediated by certain personality traits. Neuroticism and extraversion seem to be more direct mediators while agreeableness and conscientiousness are rather indirect mediators [1]. However, none of these previous studies specifically examined the link between childhood trauma and treatment-resistant depression (TRD) in the context of a geriatric population. TRD is currently defined by the failure of at least two attempted antidepressant treatments administered sequentially, at adequate dose, and for an adequate duration [8]. It can be assumed that approximately 20 to 30% of depressed patients experience TRD, as reported in Anglo-Saxon countries [9], and up to one-half of patients respond only partially [8]. TRD has been estimated to represent half of the overall treatment costs for MDD [10, 11]. Specific care packages exist for TRD in an elderly population [12]. To date, there has been little investigation examining the associations between childhood adversity and TRD. Kaplan & Klinetob [13] compared a TRD population to a population that responded successfully to antidepressants. They reported greater levels of childhood emotional abuse in the TRD group. Tunnard et al. [14] focused on TRD, although the unipolar and bipolar populations were mixed. They showed that childhood adversity was common among these TRD patients (62%) and was associated with poor clinical course, characteristics of psychosis, and suicide attempts. However, to our knowledge, no study has focused on childhood trauma and TRD in a geriatric population.

Moreover, self-esteem is associated with the clinical symptomatology and prognosis in geriatric depression. Indeed, firstly, it has been found that low self-esteem significantly increases the risk of suicidal behaviour [15]. Secondly, depressive symptoms are negatively correlated with self-esteem in this population [16]. In addition, patients with low self-esteem respond more slowly to antidepressant treatment compared to their counterparts with higher self-esteem [17].

In young adults, the association between childhood maltreatment or bullying and mental health, in particular depression, has now been well-documented in large cohorts (The Quebec Longitudinal Study of Child Development, the Avon Longitudinal Study of Parents and Children in the UK, the Great Smoky Mountains Study in the USA) [18, 19]. However, in contrast, data focusing on this association in the elderly population still remain scarce.

Thus, we posit the hypothesis that there is an association between childhood maltreatment (CM) and the severity of depression, specifically in an elderly TRD population. Moreover, given the role of personality traits as well as self-esteem in the symptomatology and course of the depressive disorder [20, 21], we postulate that personality traits and self-esteem could mediate the association between CM and the severity of depressive symptoms.

Therefore, our first objective is to assess the potential association between CM and the severity of depression. Our second objective is to investigate whether personality traits and self-esteem could influence the association between CM and the severity of depressive symptoms.

## Materials and methods

Populations: Patients were recruited for a prospective cohort (FACE-DR cohort) from the French Network of Expert Centres for Resistant Depression, consisting of 13 specialist care centres operating from within academic psychiatry departments across France [22, 23]. The recruitment for this analysis took place from July 2012 to December 2018.

The patients were clinically unresponsive to at least two successive, adequate attempts of antidepressant treatment from two different pharmacological classes, corresponding to at least stage II of the staging criteria proposed by Thase & Rush for defining TRD [24]. We selected all the cohort patients over 60 years of age for this study. Although the cut-off between geriatric and non-geriatric depression remains unclear, ranging from 60 [1, 7, 25, 26] to 65 years old [27, 28], we chose the age of 60 in order to compare our findings with those of the majority of studies focusing on the impact of CM in depressive disorders [1, 7, 25, 26]. Before participating in the full assessment, the patients were interviewed by a psychiatrist at the expert centre in order to:

- Confirm the diagnosis of TRD according to the DSM-IV (MINI) [29] criteria, with moderate to severe symptoms, the level of resistance being indicated by a Thase & Rush classification of ≥2 [24]. Since the recruitment started in 2012, we used the standard DSM-IV criteria for MDD.
- Exclude bipolar disorders, psychotic disorders, obsessive-compulsive disorders, eating disorders (with BMI < 15), somatoform disorders and mood disorders related to substance abuse or misuse.
- Inform the patient about the formal assessment procedure.

Assessment: We selected patients who were 60 or over and clinically resistant to medications as determined by the Thase & Rush staging criteria (i.e. level II) [22, 24]. The severity of depressive symptoms, CM, self-esteem and personality traits were assessed using the Montgomery-Åsberg Depression Rating Scale (MADRS) [30], the Quick Inventory of Depressive Symptomatology Self-Report (QIDS-SR) [31], the Childhood Trauma Questionnaire (CTQ) [32], the Rosenberg scale [33] and the Big Five Inventory (BFI) [34], respectively. The MADRS is a ten-item, hetero-questionnaire currently used to measure the severity of depressive episodes in patients with mood disorders. Scores range from 0 to 60 [30]. The QIDS-SR is a self-rating clinical instrument derived from the 30-item Inventory of Depressive Symptomatology. It asks 16 questions in order to assess the severity of the nine diagnostic symptom criteria used in DSM. These nine criterion-related symptoms do not assess anxious, atypical or melancholic features or other commonly associated symptoms such as pain or gastrointestinal disturbances. Scores range from 0 to 27 [31]. The CTQ is a screening tool for history of abuse and neglect. The self-report includes a 28-item scale that measures 5 types of maltreatment–emotional, physical and sexual abuse, and emotional and physical neglect [32]. The Rosenberg scale determines global self-worth by measuring both positive and negative feelings about oneself. Scores range from 0 to 30 [33]. The BFI is a self-reported inventory designed to measure the Big Five dimensions: neuroticism, extraversion, agreeableness, openness and conscientiousness. It is a 44-item multidimensional personality inventory [34].

The authors hereby confirm that all work-related procedures comply with the ethical standards of the relevant national and institutional committees on human experimentation and with the Helsinki Declaration of 1975, as revised in 2008. The assessment protocol was approved by the relevant institutional review board [French CNIL (French Data Protection Authority): DR-2015-673]. The consent obtained from study participants was written and verbal.

### Statistical analysis

Sociodemographic and clinical characteristics were presented using means and standard deviations for continuous variables, and frequency distributions for categorical variables. The chi-square test was used to compare categorical variables, and the t-test and ANOVA to compare continuous variables. Linear regression models were applied to test the association between dependent variables (MADRS, QIDS-SR) and independent variables (CTQ and subtypes, BFI subtypes, Rosenberg scale). Linear regression models were adjusted for age and sex. We used the Hayes mediation model [35] in the cross-sectional study to assess the influence of one variable on the association between CM and intensity of depressive symptoms at a specific time point. Mediation was deemed partial when indirect and direct effects were both statistically significant, and it was considered to be complete when only the indirect effect was statistically significant. Bonferroni correction was applied for multiple comparisons (CM subtypes). Statistical analyses were performed with SPSS 25.0 (IBM Corp. Released 2017. IBM SPSS Statistics for Mac, Version 25.0. Armonk, NY: IBM Corp.).

## Results

### Demographic and clinical data

**Fig 1:**
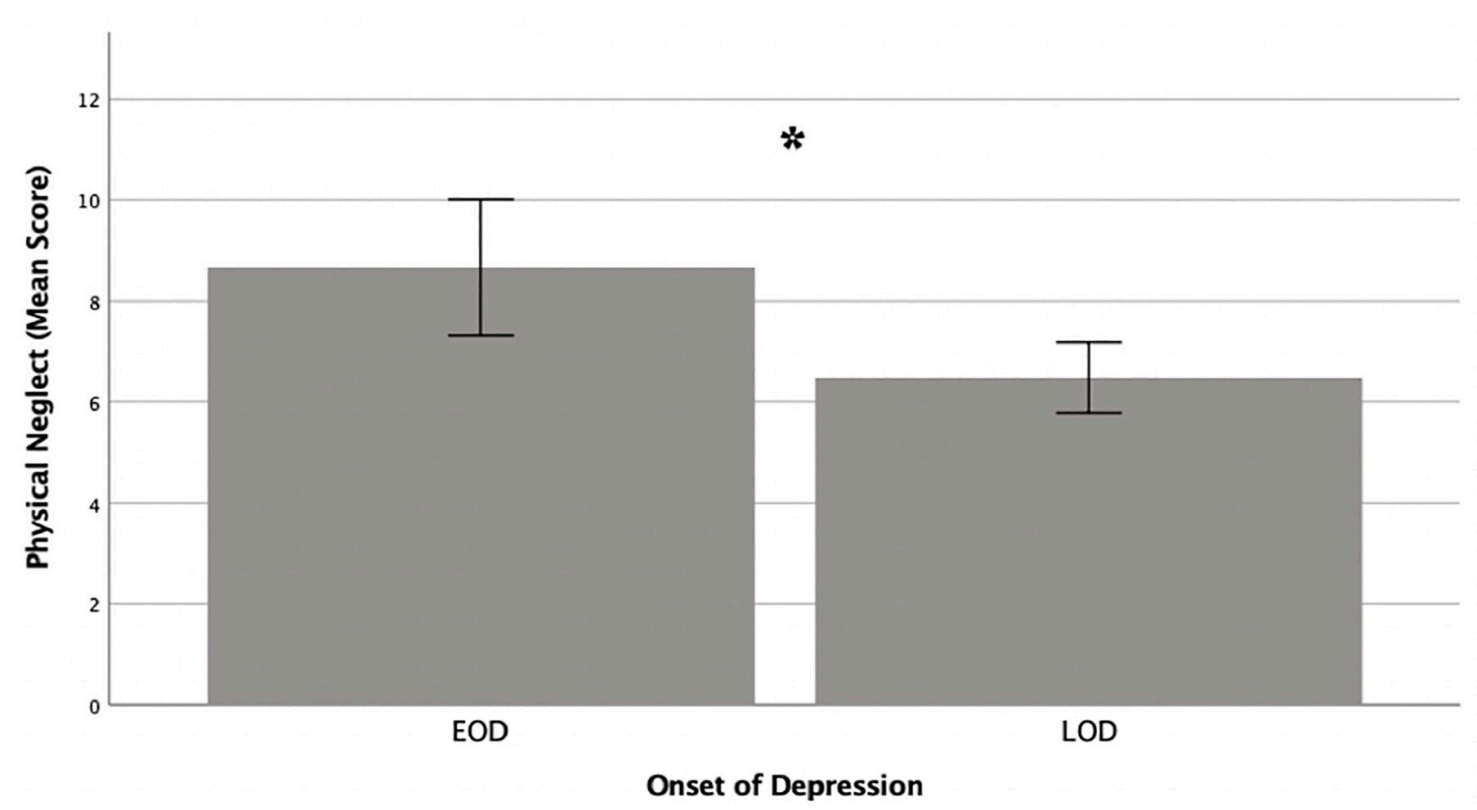
Difference of physical neglect between patients with Early Onset Depression (EOD) and Late Onset Depression (LOD).

Our study included 96 patients (Table 1) (33% of the overall cohort) with a mean age of 67.2 [standard deviation (SD): 5.7] and a majority of women (62.5%). In our sample, 82 patients (85.4%) completed the CTQ [mean score: 37.35 (SD: 9.69)]. The MADRS and QIDS-SR mean scores were high, reflecting severe depression. There was no difference in the intensity of depression symptoms on comparing the difference in terms of marital status (Table 2). A Rosenberg mean score of 22.47 (SD: 6.26) was documented. The BFI scores were: neuroticism [mean: 3.85 (SD: .63)], extraversion [mean: 2.37 (SD: .83)], agreeableness [mean: 4.14 (SD: .46)], openness [mean: 2.8 (SD: .86)] and conscientiousness [mean: 3.5 (SD: .75)]. LOD (i.e. the first episode after 60 years of age) affected 25 patients (26%). A significant difference between EOD [8.66 (SD: 4.44)] and LOD [6.48 (SD: 1.54)] was noted in relation to PN (p = 0.025) (Fig 1). However, we did not find any difference between LOD and EOD in relation to (i) other CM; (ii) the severity of depressive symptoms and (iii) self-esteem (Table 1).

**Table 1:** Population characteristics.

	General population				EOD		LOD		
	N	Min.	Max.	Mean (SD)	N	Mean (SD)	N	Mean (SD)	p
AGE, years	96	60	84	67.25 (5.67)	50 (52.1%)	65.66 (5.44)	25 (26%)	69.8 (5.77)	.**003**^**t**^
Female (%)	60 (62.5%)				34		12		.094
Marital status (%)									**.017**^**c**^
Single	8 (8.3%)				7		0		
Married	62 (64.6%)				27		20		
Separated	2 (2.1%)				2		0		
Divorced	10 (10.4%)				8		1		
Widowed	3 (3.1%)				0		2		
Educational level									.429^c^
Elementary school diploma	23 (23.8%)				10		9		
High school diploma	11 (11.5%)				4		3		
Youth training (National Vocational Qualification–Level 1, 2)	7 (7.3%)				3		1		
Business and Technology Education Council first diploma	10 (10.4%)				4		3		
High school diploma+1	3 (3.1%)				3		0		
High school diploma+2	9 (9.4%)				7		2		
High school diploma+3	6 (6.3%)				2		2		
High school dipoma+4	4 (4.2%)				4		0		
High school diploma+5	5 (5.2%)				3		1		
Doctoral Degree	6 (6.3%)				4		1		
LOD	25 (26%)								
MADRS score M0	92	8	44	28.2 (7.49)	47	29.34 (8.02)	25	27.28 (6.16)	.23^t^
QIDS-SR M0	89	1	26	16.53 (4.94)	47	17 (4.95)	23	16.43 (4.5)	.646 ^t^
Rosenberg score M0	79	10	36	22.47 (6.26)	39	22.31 (6.25)	21	22.38 (6.43)	.966 ^t^
BFI extraversion	78	1	4.25	2.37 (.83)	41	2.28 (.77)	20	2.47 (.73)	.384 ^t^
BFI agreeableness	78	3.20	5	4.14 (.46)	41	4.25 (.42)	20	3.97 (.45)	**.02** ^**t**^
BFI neuroticism	73	2	5	3.85 (.63)	40	3.96 (.53)	20	3.7 (.72)	.119 ^t^
BFI conscientiousness	73	1.22	4.89	3.5 (.75)	40	3.7 (.73)	20	3.31 (.75)	.061 ^t^
BFI openness	78	1.10	4.80	2.8 (.86)	41	2.96 (.83)	20	2.45 (.84)	**.027** ^**t**^
CTQ total score	82	25	78	37.35 (9.69)	44	38.86 (11.62)	21	35.57 (5.57)	.128 ^t^
Emotional Abuse	82	5	10	5.51 (1.07)	44	5.55 (1.23)	21	5.52 (.75)	.999 ^t^
Physical Abuse	82	5	22	11.48 (4.28)	44	12.18 (4.68)	21	11 (3.42)	.999 ^t^
Sexual Abuse	82	5	15	7.2 (2.51)	44	7.05 (2.52)	21	7.38 (2.4)	.999 ^t^
Emotional Neglect	82	5		5.35 (1.85)	44	5.43 (2.44)	21	5.19 (.68)	.999 ^t^
Physical Neglect	82	5		7.82 (3.65)	44	8.66 (4.44)	21	6.48 (1.54)	**.025** ^**t**^

BFI: Big Five Inventory

^C^: Chi2 test; CTQ: Childhood Trauma Inventory; EOD: Early Onset Depression; LOD: Late Onset Depression; M0: Month 0; MADRS: Montgomery-Asberg Depression Rating Scale; Max.: Maximum; Min.: Minimum; N: Number; QIDS-SR: Quick Inventory Depression Scale-Self Report; SD: Standard Deviation

^t^: t-test.

**Table 2:** Difference in the intensity of depression symptoms on comparing the difference in terms of marital status.

	Marital status	Depressive intensity	
MADRS (SD)	Married	28.4 (7.2)	p = 0.481^a^
	Single	25 (9.41)	
	Separated	31.5 (13.44)	
	Divorced	31.63 (7.13)	
	Widowed	29 (5.67)	
QIDS-SR (SD)	Married	16.7 (4.4)	p = 0.676 ^a^
	Single	14.25 (8.48)	
	Separated	17.5 (7.78)	
	Divorced	17.6 (4.53)	
	Widowed	15.33 (8.62)	

MADRS: Montgomery-Asberg Depression Rating Scale; QIDS-SR: Quick Inventory Depression Scale-Self Report; SD: Standard Deviation

^a^: ANOVA.

### Relationship between childhood maltreatment and depressive symptom severity

**Fig 2:**
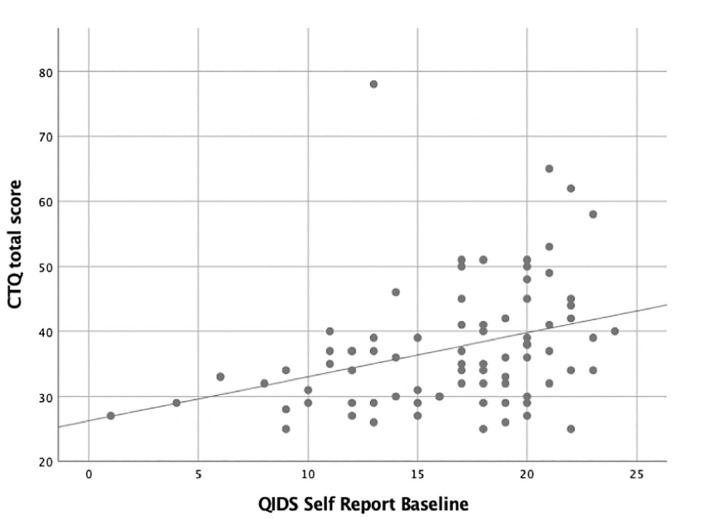
Association between Childhood trauma and intensity of self-reported depressive symptoms.

**Fig 3:**
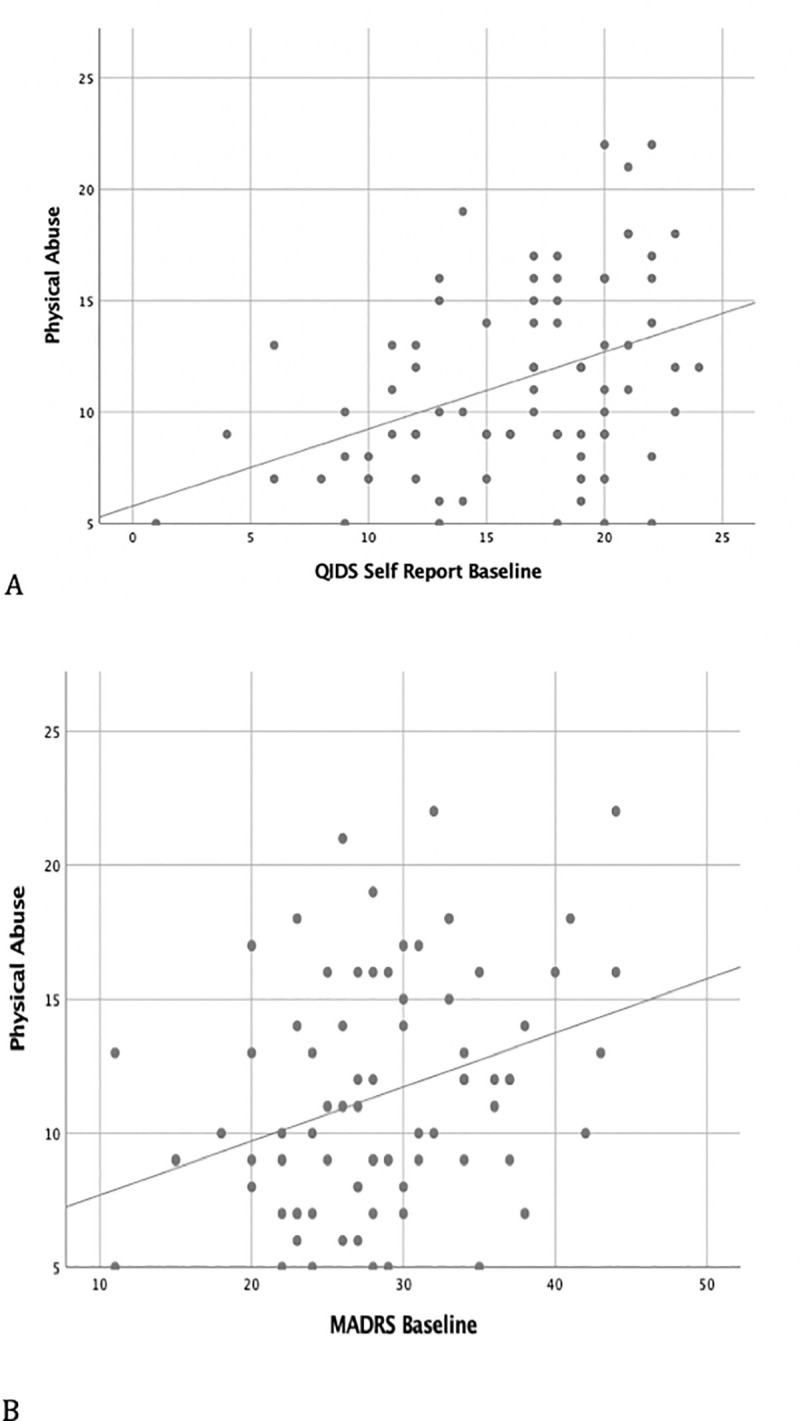
Association between Physical Abuse and intensity of depressive symptoms (A: QIDS-SR; B: MADRS).

The regression analysis found a significant positive association between CM and the intensity of depressive symptoms throughout our study sample of elderly TRD patients *(*Table 3). In an adjusted model (age and gender), CTQ scores were positively associated with MADRS (β = 0.274; p = 0.07) and QIDS-SR (β = 0.302; p = 0.005) scores (Fig 2). In relation to physical abuse (PA), we highlighted a significant association with MADRS (β = 0.304; p = 0.03) and QIDS-SR (β = 0.362; p = 0.005) scores (Fig 3). We did not, however, detect any significant association between other types of CM and the intensity of depressive symptoms (Table 3).

**Table 3:** Association between the intensity of depressive disorder and childhood trauma at baseline.

		Unadjusted		Adjusted ^a^	
		β	p-value*	β	p-value*
MADRS, score	CTQ, total score	0.3	0.04	0.274	0.07
	Emotional abuse, score	0.049	0.999	0.032	0.999
	Physical abuse, score	0.328	0.02	0.304	0.03
	Sexual abuse, score	0.263	0.09	0.251	0.11
	Emotional neglect, score	0.176	0.59	0.190	0.42
	Physical neglect, score	0.113	0.999	0.082	0.999
QIDS, score	CTQ, total score	0.338	0.002	0.302	0.005
	Emotional abuse, score	0.105	0.999	0.075	0.999
	Physical abuse, score	0.392	0.002	0.362	0.005
	Sexual abuse, score	0.199	0.37	0.184	0.45
	Emotional neglect, score	-0.037	0.999	-0.055	0.999
	Physical neglect, score	0.293	0.04	0.251	0.11

CTQ: Child Trauma Questionnaire; MADRS: Montgomery-Åsberg Depression Rating Scale; QIDS: Quick Inventory of Depressive Symptomatology;

^a^ Age and sex;

* Bonferroni corrections.

### Influence of personality traits on the relationship between childhood maltreatment and depressive symptom severity

We found no significant correlation between CM and personality traits (Table 4). Nor did we find any significant associations between distinct subtypes of CM and personality traits.

**Table 4:** Association between childhood maltreatment and personality traits.

		β	p-value*
CTQ, total score	neuroticism	0.223	0.07
	extraversion	-0.191	0.13
	agreeableness	0.052	0.68
	openness	-0.058	0.63
	conscientiousness	-0.204	0.11

CTQ: Child Trauma Questionnaire

* Bonferroni corrections.

### Influence of self-esteem on the relationship between childhood maltreatment and depressive symptom severity

**Fig 4:**
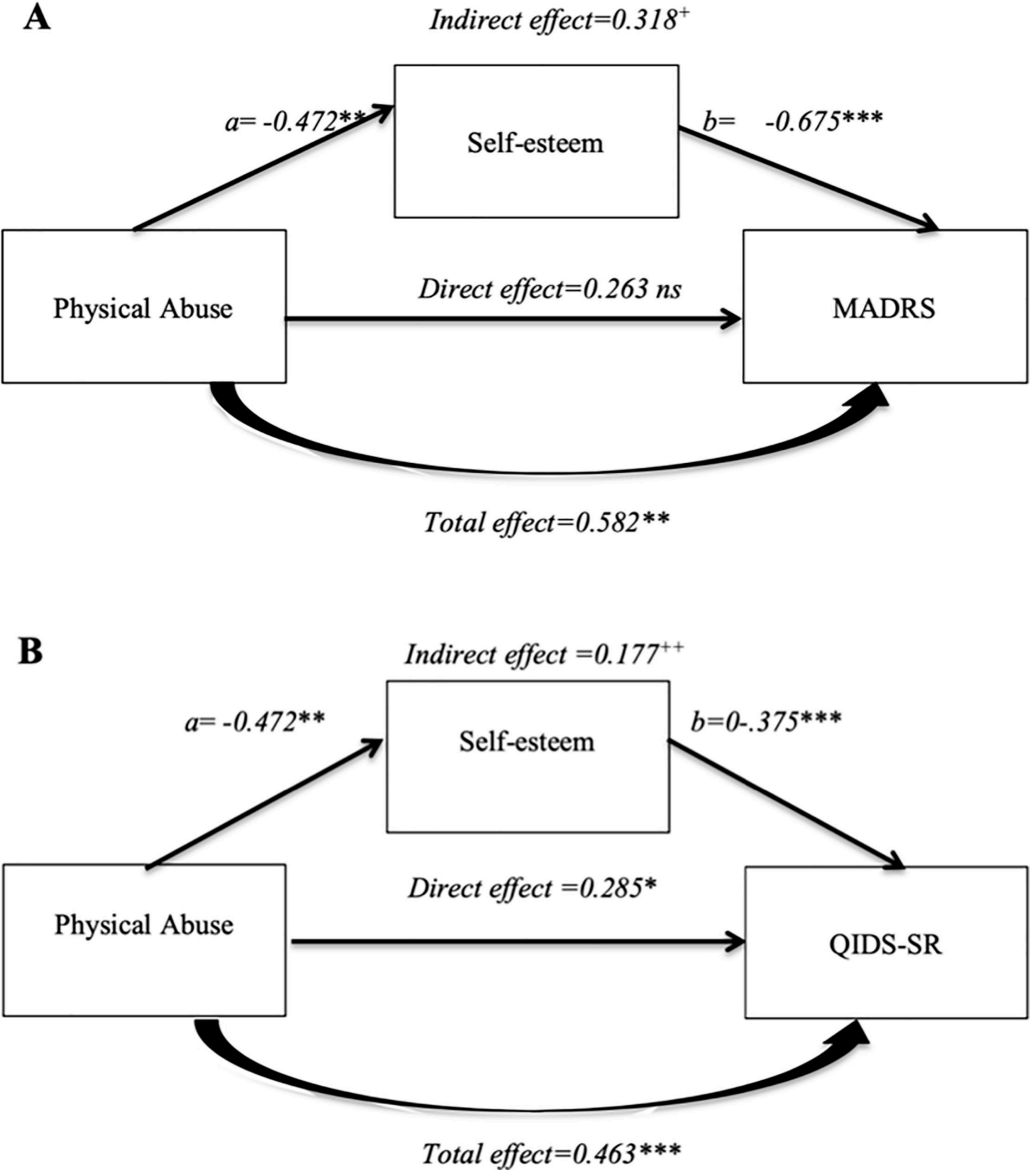
Self-esteem as mediator of physical abuse on intensity of depressive symptoms: A: MADRS; B: QIDS.

In an adjusted model, we found a negative association between CM (CTQ total) and self-esteem (β = -0.25; p = 0.036), specifically in cases reporting PA (β = -0.32; p = 0.04) (Table 5). Our objective was to assess the potential role of self-esteem on the correlation between CM (mainly PA subtype) and intensity of depression. We therefore had to consider a potential link between self-esteem and (i) CM mainly PA and (ii) intensity of depression. The association between PA and self-esteem levels (Rosenberg score) was significant (β = -0.472, p<0.01). Self-esteem levels (Rosenberg score) were associated with the intensity of depression symptoms (MADRS: β = -0.675, p<0.001; QIDS: β = -0.375, p<0.001). Therefore, we looked at a potential mediator effect of self-esteem on the correlation between PA and intensity of depression (Hayes’ model). We showed that self-esteem (Rosenberg scale) significantly mediated the effect of PA on the severity of depressive symptoms: MADRS: *b* = 0.318, 95% *BCa C*.*I*. [0.07, 0.62]; QIDS-SR: *b* = 0.177, 95% *BCa C*.*I*. [0.04, 0.37]. We documented Preacher & Kelly’s Kappa Squared values of 19.1% (*k*^*2*^ = 0.191) and 16% (*k*^*2*^ = 0.16), respectively for the two scales, indicating a moderate effect of the mediation (Fig 4). The mediation indices were found to be 0.182 [95% CI: 0.042; 0.338] for MADRS and 0.148 [95% CI: 0.031; 0.292] for QIDS-SR. Therefore, self-esteem had a moderate total mediation effect. The indirect effect of PA on depressive symptom intensity was significant (MADRS: β = 0.318, 95%CI [0.07; 0.624], QIDS: β = 0.177: 95%CI [0.036; 0.377]). The direct effect of PA on depression symptom severity was a positive, but not statistically significant, association when focusing on MADRS (β = 0.263, p = 0.132). However, the correlation was both positive and significant when focusing on QIDS-SR (β = 0.285, p < 0.05). The full model effect of PA on intensity of depression symptoms was significant (MADRS: β = 0.582, p<0.01; QIDS: β = 0.463, p<0.001) (Fig 4). Self-esteem appeared to mediate the association between PA in childhood and depression symptom severity.

**Table 5:** Association between self-esteem and childhood trauma at baseline.

		Unadjusted		Adjusted ^a^	
		β	p-value*	β	p-value*
Rosenberg, score	CTQ, total score	-0.25	0.035	-0.25	0.036
	Emotional abuse, score	0.11	0.99	0.11	0.99
	Physical abuse, score	-0.31	0.04	-0.32	0.04
	Sexual abuse, score	-0.27	0.12	-0.27	0.1
	Emotional neglect, score	-0.06	0.999	-0.05	0.999
	Physical neglect, score	-0.12	0.999	-0.11	0.999

CTQ: Child Trauma Questionnaire; MADRS: Montgomery-Åsberg Depression Rating Scale; QIDS: Quick Inventory of Depressive Symptomatology; STAI: State-Trait Anxiety Inventory;

^a^ Age and sex

* Bonferroni corrections.

## Discussion

To our knowledge, this is the first study in a geriatric TRD population to document an association between CM, specifically related to PA, and the intensity of depressive symptoms, using either self- or hetero-evaluations. Moreover, we found that self-esteem can influence the association between CM and depressive symptom severity scored with MADRS and partially with QIDS-SR. Our findings suggest therefore that low self-esteem is an important factor in geriatric depression, especially in individuals with a history of PA.

To date, the only available study focusing on the correlation between CM and TRD is Tunnard *et al*. [14]. However, this study was not conducted in the field of geriatric depression since the recruited population was particularly heterogeneous and included both unipolar and bipolar patients. They reported that CM is common, affecting up to 62% of TRD patients. However, they failed to show any significant correlation between CM and clinical severity.

Moreover, self-esteem has been shown to be associated with geriatric depression symptoms [15] and prognosis [17]. Numerous studies have documented a strong positive correlation between CM and low self-esteem [36–38]. This association could be explained by the fact that early maltreatment can negatively affect the overall cognitive, social and emotional development of children. Also, it is well-established that low self-esteem and depression are closely linked [36–38]. Finally, self-esteem was found to peak in people in their fifties or sixties before decreasing dramatically thereafter. Two explanations for this decline have been put forward [39, 40]. The first refers to the loss of elements associated with self-esteem, ranging from socioeconomic positions or social roles due to retirement to abilities such as physical and cognitive performance, etc. The second relates to profound changes in attitudes toward oneself in that elderly people tend to accept their limitations as they get older, which leads them to take a more modest view of themselves.

In contrast to previously published studies [1, 6, 7], we found no significant correlation with personality traits in our work focusing specifically on TRD in the geriatric population. Our study sample was selected from highly specialised care centres in contrast to other published studies that focused on samples from the general population [1, 6, 7].

There were some limitations. Firstly, our sample was smaller than that of other studies carried out in geriatric populations [1, 6, 7]. This could account for the lack of correlation between CM and personality traits. Moreover, we did not compare our results to a control group of depressed patients showing no resistance to treatment, in order to assess whether mediation through self-esteem is closely related to TRD. In addition to this, the cut-off age we used for geriatric depression was relatively young (60 years of age and above). However, this cut-off is the same as that used in other studies [1, 6, 7]. In addition, the diagnoses of EOD and LOD were determined retrospectively. This may have introduced a bias and may explain the lack of data in relation to the first major depressive episode. There could also be a recall bias regarding CTQ. Indeed, a recall bias, or greater likelihood of reporting exposure in participants with MDD, has been highlighted due to negative bias in autobiographical memory [41]. Moreover, the mediation analysis should be interpreted with caution given the fact that this was a cross-sectional study. Finally, the involvement of other factors such as cognitive decline and traumatic events occurring in adulthood, which could be linked to the intensity of depressive symptoms, at least in part, would require further investigation in research carried out in geriatric TRD populations.

Despite the need for future studies to confirm our findings, it seems important to take the mediation effect of self-esteem into account in routine clinical practice. Indeed, relevant data indicate the positive effect of psychotherapy, such as cognitive-behavioural therapy (CBT), on low self-esteem [42–46]. Therefore, CBT focused on self-esteem could be particularly useful for the management of TRD, especially in patients with early negative life experiences. In fact, given that higher self-esteem is significantly correlated with better treatment responses, increased self-esteem could improve the medical care outcome [47, 48].

## Conclusion

We highlighted a correlation between CM mainly related to PA and the intensity of depressive symptoms in a geriatric TRD population. Our findings must be confirmed in well-designed prospective studies on larger pathological populations. However, these results underpin the potential relevance of CBT predominantly focused on self-esteem as an add-on psychological intervention useful for managing TRD in geriatric populations, especially in cases who experienced CM.

## Supporting information

S1 Data (XLSX)

## Abbreviations

BFI: Big Five Inventory
BMI: Body Mass Index
CM: Childhood Maltreatment
CTQ: Childhood Trauma Questionnaire
DSM: Diagnostic and Statistical Manual of Mental disorders
EA: Emotional Abuse
EN: Emotional Neglect
EOD: Early Onset Depression
FACE-DR: French Network of Expert Centres for Resistant Depression
LOD: Late Onset Depression
MADRS: Montgomery-Åsberg Depression Rating Scale
MDD: Major Depressive Disorder
MINI: Mini International Neuropsychiatric Interview
PA: Physical Abuse
PN: Physical Neglect
QIDS-SR: Quick Inventory of Depressive Symptomatology-Self Report
SA: Sexual Abuse
SD: Standard Deviation
TRD: Treatment Resistant Depression
